# Heath management app use in Parkinson’s disease and quality of life during the COVID-19 pandemic

**DOI:** 10.1038/s43856-023-00246-4

**Published:** 2023-02-11

**Authors:** Yilin Tang, Xiaoniu Liang, Bo Shen, Jiawei Wang, Bastiaan R. Bloem, Jianjun Wu, Jian Wang

**Affiliations:** 1 Department of Neurology and National Research Center for Aging and Medicine & National Center for Neurological Disorders, State Key Laboratory of Medical Neurobiology, Huashan Hospital, Fudan University, Shanghai, China; 2 College of Arts and Science, Vanderbilt University, Nashville, TN 37212 USA; 3 Department of Neurology, Radboud University Medical Centre, Donders Institute for Brain, Cognition and Behaviour, Centre of Expertise for Parkinson and Movement Disorders, Nijmegen, The Netherlands

**Keywords:** Parkinson's disease, Epidemiology

## Abstract

**Background:**

Social distancing during the COVID-19 pandemic affected follow-up visits and medication availability for patients with Parkinson’s disease (PD). As a promising strategy to deal with these challenges, the implementation of health management smartphone apps was accelerated. However, whether more intense use of such apps could improve the quality of life (QoL) for PD patients during the COVID-19 pandemic was unknown.

**Methods:**

Using a PD management app, this observational study assessed changes in QoL, as determined by PD Questionnaire 8 (PDQ-8), among PD patients before (Jan 20, 2019 to Oct 6, 2019) and after the beginning of the COVID-19 lockdown (Jan 20, 2020 to Oct 6, 2020). According to adherence to use of the app, participants were divided into low adherence, moderate adherence, and high adherence groups. A total of 4979 PD patients registered in the app, and 226 PD patients were enrolled, including 57 patients with low adherence, 112 with moderate adherence and 57 with high adherence. A generalized linear model was used to evaluate the change of PDQ-8 scores across these three different adherence groups.

**Results:**

After the COVID-19 lockdown (1-year follow-up), the PDQ-8 scores are reduced by 0.8 (95% CI, 0.3–1.4) in all participants (*P* = 0.004). After adjustment for age, gender, education, disease duration and levodopa equivalent dose, PDQ-8 scores significantly less reduced in the high adherence group (0.3; 95% CI, 0.6–1.2) compared to the low adherence (1.9; 95% CI, 0.7–3.1) (*P* = 0.040) and moderate adherence groups (0.6; 95% CI, 0.2–1.3) (*P* = 0.012).

**Conclusions:**

A health management smartphone-based app might be a way to both measure and improve QoL among PD patients, provided that sufficient adherence is achieved.

## Plain language summary

The COVID-19 pandemic had an enormous impact on the lives of people living with Parkinson’s disease (PD), given social distancing measures, and reduced access to healthcare. As a way to mitigate this, the use of health management apps was accelerated. However, there is a lack of studies on whether the use of such apps could over time affect the quality of life of people living with PD. Here, we analyzed changes in the quality of life of people living with PD using a health management app, before and after social distancing. We found that patients with high adherence to use of the app experienced a lower reduction of their quality of life. Using a health management smartphone app represents a novel approach that might help patients with PD improve their quality of life.

## Abstract (web summary)

Tang, Liang, Shen, et al. assessed whether the use of a health management app by people with Parkinson’s disease before and after a COVID-19 lockdown in China had an impact on their quality of life (QoL). They show that these social distancing measures reduced the QoL overall, but the reduction was less pronounced if adherence to the app was high.

## Introduction

The ongoing COVID-19 pandemic has an enormous impact on the lives of many people worldwide. Some national governments have introduced extensive ‘lockdown’ measures including social distancing to contain the spread of transmission of COVID-19. Individuals with a chronic disease, including Parkinson’s disease (PD), are particularly vulnerable to such restrictions. There is limited capacity for follow-up visits, a restricted medication availability, less options to engage in regular physical activity, including inability to participate in exercise classes, and increased levels of psychological distress^1,2^.

Mobile health interventions, such as videoconferencing or smartphone applications (apps), have been advocated as promising strategies to assist in the management of PD^3^. These telemedicine tools have the potential to ascertain routine follow-up visits remotely, organize new subspecialty consultations, perform remote research visits, and also to deliver interventions remotely, including psychotherapy, social services, rehabilitation, and education. The merits of telemedicine interventions are supported by a small but growing body of evidence^4–10^. Potential benefits include cost savings, a reduction in travel distance, and a reduction in time spent attending appointments. However, most studies using telemedicine tools for patients with PD were developed only for specific self-management properties such as medication diary, remote consultation, or exercise.

Currently, the COVID-19 crisis is catalyzing the use of mobile health interventions, which have now become an attractive alternative route of delivering care to PD patients while adhering to the social distancing measures^11,12^. To our knowledge, no study has tested whether the use of telemedicine strategies could affect a clinically important outcome—namely quality of life (QoL)—in PD patients during the COVID-19 pandemic. In this observational study, we first assess the change in QoL for PD patients before and after the COVID-19 lockdown using a novel smartphone-based app, that is designed to remotely cover most of the aspects regarding the management of PD^13^. Then, we seek to test whether more intense use of the app would be associated with better QoL during the COVID-19 pandemic.

## Methods

### Study Population

PD was diagnosed by a specialist in movement disorders according to the Movement Disorder Society Clinical Diagnostic Criteria during an in-clinic evaluation^14^. PD patients who owned IOS or Android smartphones were invited to download an app developed by our group as a not-for-profit effort to support the management of PD remotely (Pawei—which is Chinese for “for Parkinson’s disease”). Details about the app are provided below. Participants were provided with instructions on how to use the app. After completing the registration, the app directs the participants to an informed consent page, where the participants can either “agree” or “decline” their participation. The study was approved by the Institutional Review Board of the Huashan Hospital (approval number: KY2016-214). The consents are provided by all patients, but given the remote nature of the application does not require a physical signature. The study is registered at the ClinicalTrials.gov database under registration number NCT03649503. Since social distancing began officially in China on Jan 20, 2020, inclusion criteria included, (i) a diagnoses of idiopathic PD, (ii) access to a smartphone on a daily basis at home, (iii) completed both a self-evaluation prior to introduction of these measures (baseline scores, between Jan 20, 2019 and Oct 6, 2019) and a follow-up self-evaluation one year later (between Jan 20, 2020 and Oct 6, 2020) (detailed workflow in Supplementary Fig. 1). Key exclusion criteria included, (i) suspected Parkinsonism due to causes other than idiopathic PD, (ii) a diagnosis of dementia, stroke, epilepsy, encephalitis, traumatic brain injury, malignancies, or severe psychiatric illness (as recorded in patient case file).

Following consent, participants are asked to complete self-evaluation questionnaires, at baseline and at least once each season (at 90-day intervals) for regular evaluation. At each evaluation, participants completed the Parkinson Disease Questionnaire 8 (PDQ-8, the original score ranges from 0 to 32)^15,16^, Geriatric Depression Scale 15 (GDS-15)^17^, and Movement Disorder Society-Sponsored Revision of the Unified Parkinson’s Disease Rating Scale (MDS-UPDRS) IB and II^18^. Furthermore, patients were instructed to complete information about previous and current medication use. The dosage of anti-parkinsonian drugs was converted into a total daily LED^19^.

### The PD remote management app

In April 2017, we launched a platform for iOS and Android smartphone applications for Chinese patients and doctors^13^. At the time of the start of this present study (Jan 20, 2019), 126 hospitals across China had joined this platform.

The app consisted of the following features (Supplementary Fig. 2):

1. Episodic delivery of various self-evaluation questionnaires;
2. Establishing remote communication between patients and physicians, including post-intervention feedback;
3. Offering educational material about PD management (articles, audios and videos) and illustrative educational case histories;
4. Evaluating disease severity based on uploaded video and voice recordings (self-recorded by patients at home).

### Adherence to use the app

We quantified the frequency of using the app based on three elements: (1) the number of times per month when patients accessed the educational materials; (2) the number of remote patient-physician consultations; and (3) the number of completed episodic self-evaluation questionnaires. The raw number of those times was transformed into a z-score by subtracting the mean number of times from the participant, and then dividing the difference by the standard deviation of the number of times according to the following formula:

Z-score = (number of times − mean)/SD.

The mean of three z-scores for each part was used as the compound score of app usage frequency. Using the distribution of the compound score, the following definitions were created: low adherence: compound score below the 25th percentile; moderate adherence: compound score ≥25th percentile and ≤75th percentile; and high adherence: compound score greater than 75th percentile.

### Outcomes

The primary outcome was change in QoL, as determined by PDQ-8 before and after the COVID-19 lockdown.

### Statistical analysis

Categorical variables were expressed as frequencies (%), and continuous variables were expressed as the mean ± standard deviation (SD). Across the three groups (low adherence, moderate adherence, and high adherence), the Chi-squared test was used for comparing the categorical variables, and the Kruskal–Wallis test or one-way ANOVA for comparing continuous variables. Wilcoxon signed-rank test was used for two paired groups. The generalized linear model (GLM) was used to assess the association between PDQ-8, GDS-15, MDS-UPDRS IB and II scores and different adherence group among the participants. Unadjusted models were not adjusted for any covariate, and adjusted models were controlled for age, gender, education, disease duration, and LED at baseline. Spearman rank correlation was used to assess the correlation between the compound score of app usage frequency and baseline and follow-up information. Two-tailed *p* values are presented. Differences were considered statistically significant at *P* < 0.05. The data analysis was conducted by SAS 9.4 (SAS Institute Inc., Cary, NC, USA).

### Reporting summary

Further information on research design is available in the Nature Research Reporting Summary linked to this article.

## Results

### Participants

Since its inception, a total of 4979 PD patients registered at the app and agreed to share their data. 2446 PD patients completed at least one regular self-evaluation. 226 PD patients who completed baseline self-evaluation before social distancing, and the 1-year follow-up self-evaluation were enrolled in this study (Supplementary Table 1).

### Baseline characteristics

Clinical characteristics are described in Table 1.

**Table 1:** Baseline and 1-year follow-up clinical features of participants.

Variables	Total (*N* = 226)	Low adherence (*N* = 57)	Moderate adherence (*N* = 112)	High adherence (*N* = 57)	*P* value^a^
No. of reading the educational materials, mean (SD)	9.1 (15.9)	4.8 (4.4)	7.4 (8.0)	14.0 (24.4)	0.002
No. of self-evaluation, mean (SD)	17.7 (12.2)	11.5 (8.6)	15.1 (8.1)	29.2 (14.4)	<0.001
No. of consultations, mean (SD)	7.0 (4.0)	3.5 (1.5)	6.5 (2.0)	11.0 (3.4)	<0.001
Z-score of app usage frequency, mean (SD)	0.0 (0.5)	−0.5 (0.2)	−0.1 (0.1)	0.7 (0.5)	<0.001
Age, year, mean (SD)	60.2 (11.3)	60.7 (12.2)	60.2 (11.8)	59.4 (9.4)	0.829^b^
Age at onset, year, mean (SD)	56.4 (11.2)	57.6 (12.1)	56.0 (11.6)	56.0 (9.4)	0.655^b^
Disease duration, year, mean (SD)	2.6 (3.2)	2.3 (3.0)	3.0 (3.5)	2.2 (2.9)	0.081
BMI, kg/m^2^, mean (SD)	24.5 (5.7)	24.9 (8.0)	24.2 (5.1)	24.6 (4.8)	0.711
Education, year, mean (SD)	12.2 (3.9)	13.1 (3.1)	11.4 (4.1)	12.9 (4.0)	0.139^b^
Gender, male, *n* (%)	129 (57.1)	29 (50.9)	69 (61.6)	31 (54.4)	0.368^c^
Custom Hand, right hand, *n* (%)	33 (82.5)	8 (100.0)	15 (71.4)	10 (90.9)	0.134^c^
Baseline self-evaluation					
PDQ-8, score, mean (SD)	9.6 (7.2)	9.4 (6.3)	10.3 (7.9)	8.3 (6.4)	0.370
GDS-15, score, mean (SD)	7.1 (2.6)	7.4 (3.1)	7.5 (2.6)	6.5 (2.1)	0.263
MDS-UPDRS IB, score, mean (SD)	7.1 (5.1)	7.3 (4.8)	7.7 (5.4)	6.3 (4.8)	0.413
MDS-UPDRS II, score, mean (SD)	11.6 (7.0)	11.6 (6.0)	11.9 (7.2)	11.4 (7.6)	0.843
LED, mg/day, mean (SD)	440.6 (325.4)	391.6 (272.7)	449.5 (316.3)	472.8 (387.1)	0.384
1-year follow-up self-evaluation					
PDQ-8, score, mean (SD)	10.4 (7.4)	11.3 (7.3)	10.9 (7.8)	8.7 (6.6)	0.096
GDS-15, score, mean (SD)	7.1 (2.6)	8.6 (2.5)	7.0 (2.7)	6.4 (2.3)	0.054
MDS-UPDRS IB, score, mean (SD)	9.3 (6.6)	12.8 (7.7)	9.8 (6.8)	6.3 (4.3)	0.095
MDS-UPDRS II, score, mean (SD)	13.9 (10.4)	19.3 (9.8)	15.0 (11.4)	8.7 (6.2)	0.022
LED, mg/day, mean (SD)	626.6 (340.8)	576.2 (265.7)	613.7 (331.5)	702.0 (411.8)	0.128

*BMI* body mass index, *PDQ-8* Parkinson Disease Questionnaire 8, *GDS-15* Geriatric Depression Scale 15, *MDS-UPDRS* Movement Disorder Society-Sponsored Revision of the Unified Parkinson’s Disease Rating Scale, *LED* levodopa equivalent dose.

^a^Comparison among the three groups with low adherence, moderate adherence, and high adherence.

^b^The continuous variables were compared among the three groups by one-way ANOVA test. The other continuous variables were compared among the three groups by Kruskal–Wallis test.

^c^The Chi-squared test was used for comparing the categorical variables.

Based on the compound score of app usage frequency, 57 patients were classified as having manifested a low adherence, 112 patients as moderate adherence, and 57 patients as high adherence. The clinical characteristics were well balanced among groups at baseline.

We used spearman rank correlation to assess the correlation between the compound score of app usage frequency and baseline characteristics. As presented in Supplementary Table 2, we found a significant but modest correlation between the compound score and baseline LED (*r* = +0.14, *P* = 0.047). We observed no correlation between the compound score and other baseline characteristics.

### Quality of life

At baseline, there was no significant difference in PDQ-8 scores across the three groups (*P* = 0.370) (Table 1).

After the COVID-19 lockdown (1-year follow-up), the mean (SD) PDQ-8 score increased by 0.8 (4.1) (*P* = 0.004) in all the participants. The mean (SD) PDQ-8 score increased by 1.9 (4.6) (*P* = 0.004) in the low adherence group, 0.6 (4.0) (*P* = 0.137) in the moderate adherence group, and 0.3 (3.5) (*P* = 0.833) in the high adherence group. This change in PDQ-8 score over time differed significantly across the three groups (Table 2). After adjustment for age, gender, education, disease duration, and LED, the increase in PDQ-8 scores in the high adherence group was less than the low adherence group (*P* = 0.040) and the moderate adherence group (*P* = 0.012) (Table 2).

**Table 2:** Change in PDQ-8, GDS-15, MDS-UPDRS IB and II scores by adherence group.

Variables	Total (*N* = 226)	Low adherence (*N* = 57)	Moderate adherence (*N* = 112)	High adherence (*N* = 57)	*P* value^a^	*P* value (unadjusted effect Estimate)^c^			*P* value (adjusted effect estimate)^c^		
						L vs M	L vs H	M vs H	L vs M	L vs H	M vs H
PDQ-8 change score, mean (SD)	0.8 (4.1)	1.9 (4.6)	0.6 (4.0)	0.3 (3.5)	0.004	0.015^*^	0.012^*^	0.414	0.994	0.040^*^	0.012^*^
GDS-15 change score, mean (SD)	0.3 (1.9)	1.3 (2.7)	0.2 (2.0)	0.1 (1.6)	0.596^b^	0.667	0.529	1.000	0.180	0.039^*^	0.349
MDS-UPDRS IB change score, mean (SD)	1.3 (3.7)	2.0 (3.5)	0.8 (3.7)	1.7 (3.9)	0.906	0.926	0.893	0.989	0.524	0.823	0.392
MDS-UPDRS II change score, mean (SD)	1.0 (5.3)	2.7 (7.4)	1.7 (5.6)	−0.1 (4.6)	0.738	0.986	0.945	0.714	0.780	0.553	0.050

*PDQ-8* Parkinson Disease Questionnaire 8, *GDS-15* Geriatric Depression Scale 15, *MDS-UPDRS* Movement Disorder Society-Sponsored Revision of the Unified Parkinson’s Disease Rating Scale, *L* Low Adherence, *M* Moderate Adherence, *H* High Adherence.

^*^P < 0.05.

^a^Comparison among the three groups with low adherence, moderate adherence, and high adherence.

^b^The continuous variables were compared among the three groups by one-way ANOVA test. The other continuous variables were compared among the three groups by Kruskal–Wallis test.

^c^The generalized linear model was used to assess the association between PDQ-8, GDS-15, MDS-UPDRS IB and II scores, and different adherence group.

*P* Value for unadjusted effect estimate was the result that the models didn’t control for any covariates; *P* Value for adjusted effect estimate was the result that the models controlled for age, gender, education, disease duration, and levodopa equivalent dose.

### Other non-motor self-evaluations

At baseline, a comparison of GDS-15 score, MDS-UPDRS IB and II score showed no significant difference (Table 1). After adjustment for age, gender, education, disease duration and LED, there was no significant difference in change of MDS-UPDRS IB and MDS-UPDRS II score across the three groups. However, the increase in GDS-15 score in the high adherence group was less than the low adherence group (*P* = 0.039) (Table 2).

## Discussion

Our study showed an expected worsening in QoL (reflected by higher PDQ-8 scores) in PD patients after the COVID-19 lockdown. Additionally, we show that PD patients with high adherence to use of the PD management app experienced less worsening of their QoL compared to those with moderate and low adherence.

The ongoing COVID-19 pandemic has an enormous impact on PD patients. Social distancing has affected follow-up visits and medication availability. The other highly disconcerting consequences of the pandemic include increased levels of stress and a marked reduced physical exercise. This is associated with worsening of both motor and non-motor symptoms^2,20–24^. QoL is a crucial outcome indicator, and being able to maintain or even improve QoL is an important objective of treatment and care in PD patients. A previous study investigated the impact of the COVID-19 pandemic on QoL of PD patients. The findings suggested that PD patients had significantly worse PDQ-39 dimensions compared with controls^1^. However, no prior study has compared the QoL in PD directly before and after COVID-19 outbreak through smartphone-based app. In our study, we showed that the PDQ-8 scores significantly worsened after the COVID-19 lockdown, indicating that QoL became more jeopardized. However, we cannot exclude the possibility that the worsening of QoL resulted primarily from progression of the underlying disease.

This COVID-19 pandemic calls for the introduction of better self-management strategies that can help patients to better deal with the challenges of social distancing and the other consequences of this crisis. The implementation of telemedicine solutions, including apps designed for the smartphone, was accelerated during this crisis^11,12^. Whether more intense use of smartphone apps could minimize the possible negative impact of COVID-19 pandemic was hitherto unknown. In this study, we explored the association between adherence of using a self-developed PD management app and QoL among PD patients. Our results demonstrate that PD patients with high adherence (and who therefore utilized more services that were delivered via the app) exhibited less worsening of QoL than the moderate and low adherence group, an effect that persisted after correction for confounding variables. We hypothesize several reasons why a smartphone-based remote management app might improve QoL for PD patients. First, being grounded at home induces or aggravates neuropsychiatric symptoms such as depression^2,23,25^. The smartphone-based app may have helped to diminish the feeling of social isolation and alleviate depression by providing remote support for people living with PD. An argument in favor of this explanation is the fact that PD patients with high adherence exhibited less worsening of the GDS-15 score than the low adherence group in this study. However, since the mean change in the GDS-15 score between low and high adherence group were modest, whether use of apps have an impact on depression warrant further investigation. Second, the app may have promoted patient self-management, enhanced treatment adherence, and improved the quality of the clinical consultations. In this way, patients may have become active co-actors in the management of their disease, gaining better and more personalized treatment, which is particularly important in a heterogeneous disorder like PD. Third, the educational material about PD management and best practice examples on how to facilitate daily life may have further helped to support patients in their self-management, and also facilitated the remote communication with physicians by minimizing the knowledge gap between doctors and patients. We suspect that the improvement in QoL might have resulted from a mix of these various services, rather than from a single functionality provided by app. However, our observational studies can only reveal association between smartphone application use and QoL, a designed experiment is needed to establish a causal inference.

It is also important to address the fact that many patients did not adhere faithfully to using the app. Out of a total of 4979 registered PD patients, only 49.1% patients completed at least one regular self-evaluation. Several other studies also reported a high dropout rate amongst users of smartphone apps: 26% of apps are used only once and 74% of apps are not used more than 10 times^26^. Methods to increase participants’ engagement with smartphone applications should be an important topic in future mobile health studies. Strategies to improve retention may include developing a simpler, more user-friendly application, providing patients with instant feedback, adding an appropriate incentive into the application, such as the ability to conveniently book clinical appointments, or possibly even financial incentives. Finally, use of briefer questionnaires may be helpful to improve compliance. The extensive 39-item Parkinson’s disease Questionnaire (PDQ-39) is the most widely used PD-specific health-related QoL questionnaire. To provide greater convenience for patients, the PDQ-8 could be used, which is a briefer version of the PDQ-39 and which can assess QoL more quickly, with less burden for the respondents^15^. The validity and reliability of the PDQ-8 have been demonstrated in several studies^16,27^.

This study has several limitations. First, participants were people who owned IOS or Android smartphones and thus were not representative of the broader PD population. Further studies with PD patients without own smartphones remain needed. Second, we corrected for various potential confounding factors impacting on QoL, including age, gender, education, disease duration, and LED. Nonetheless, we cannot fully exclude a possible influence of other factors that were not evaluated by this app, such as UPDRS-III scores, falls, the existence of caregivers, which might also impact on QoL. Obtaining information about these potentially important factors will be necessary in future studies to minimize potential biases. Finally, the present observation lasted for one year; although this is reasonable for a first orienting studies such as ours, more work remains needed to monitor the effect of a smartphone-based remote management app in the long term. It will also be interesting to examine whether any observed benefits persist after participants stop using the app.

## Conclusion

In this study, we found that PD patients with high adherence to use of the PD management app experienced less worsening of their QoL. Our results suggest that a smartphone-based app might be a novel way to both measure and improve QoL among PD patients during the COVID-19 pandemic. Further work in a larger sample size and longer periods of time is recommended to determine whether the benefits of the smartphone app are maintained.

## Supplementary information

Supplementary Information

Reporting Summary

## Data Availability

The data are available from Huashan Hospital, but restrictions apply to protect the privacy of individuals, so the data are not publicly available. Data are, however, available from the authors upon reasonable request and with permission from Huashan Hospital.
