# Supplementary material for: Heath management app use in Parkinson’s disease and quality of life during the COVID-19 pandemic
Source: Commun Med (Lond). 2023 Feb 11;3:23. doi: 10.1038/s43856-023-00246-4 (PMC9919748; doi:10.1038/s43856-023-00246-4)
Supplement: Supplementary file 1 — Supplementary Information [file 43856_2023_246_MOESM1_ESM.pdf]

Supplementary Information

Supplementary Figure 1. Flowchart of study participants

Supplementary Figure 2. The app functionality for PD patients

Supplementary Table 1. Baseline demographics of the app users

Supplementary Table 2. The correlation between the compound score of app usage frequency and baseline and follow-up information

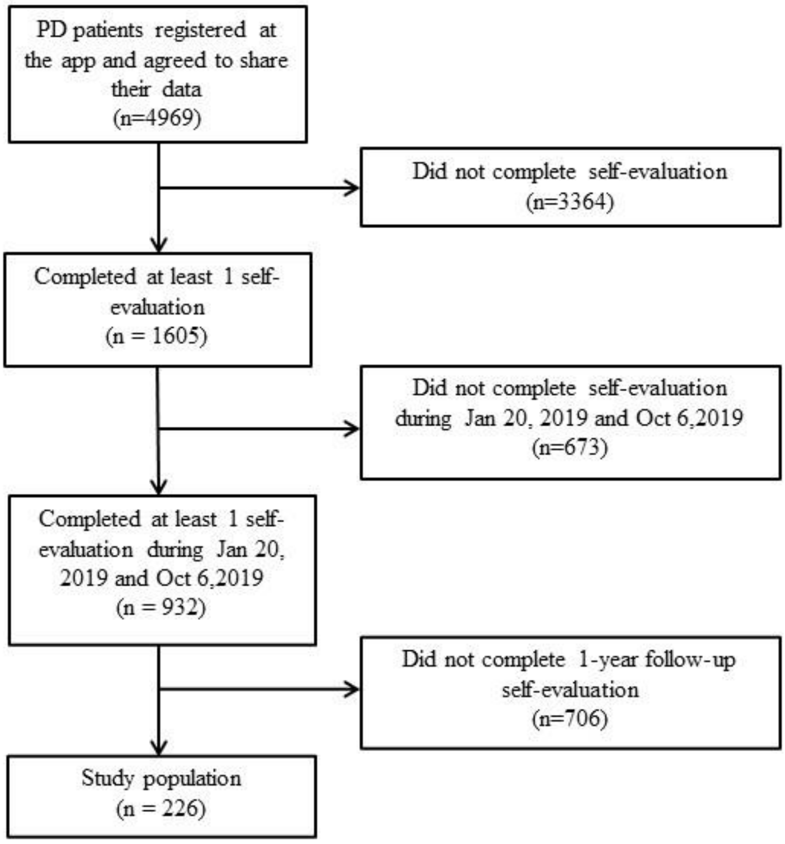

Supplementary Figure 1. Flowchart of study participants

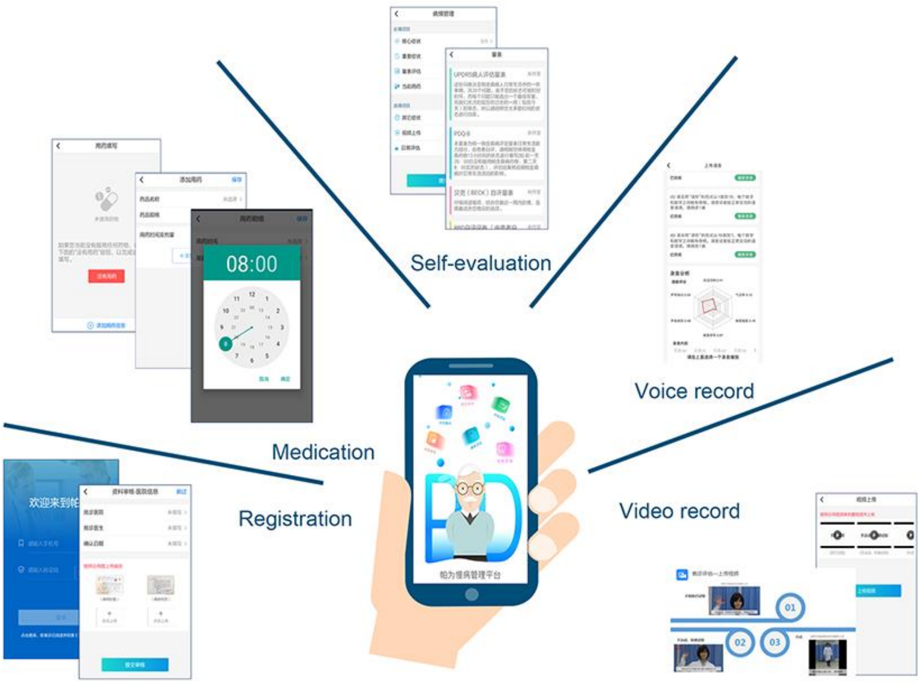

Supplementary Figure 2. The app functionality for PD patients. The primary modules of this app for patients include registration, medication, self-evaluation, voice recordings, video recordings and other functions. Registration**:** Participants register by entering phone number and verification code; Medication: Previous and current treatment information (duration and dosages in PD drugs, whether use drugs induced parkinsonism or other drugs) is collected. This information allows the clinician to review the current and past medication plans and register a new one if required; Self-evaluation: Participants are asked to complete self-evaluation questionnaires, at baseline and at least once each season (at 90-day intervals) for regular evaluation. Data presented to doctor can provide a complete assessment of the patient's condition, including motor and non-motor symptoms; Voice recordings: Participants are requested to record their voice. Disease severity is evaluated based on uploaded voice recordings; Video recordings: Participants are asked to perform video-recorded UPDRS-III test after the imitation of a visual cue on a mobile screen. Motor symptoms are judged by clinicians based on the video recordings.

Supplementary Table 1. Baseline demographics of the app users

Variables	All (N=4979)	Completed at least one self-evaluation (N=2446)	Study population (N=226)
Age, year, mean (SD)	61.8 (11.3)	60.8 (11.6)	60.2 (11.3)
Gender, male, n (%)	2681 (54.0)	936 (54.9)	129 (57.1)
Education, year, mean (SD)	10.9 (4.7)	11.6 (4.4)	12.2 (3.9)
Disease duration, year, mean (SD)	\	3.1 (6.3)	2.6 (3.2)

Supplementary Table 2. The correlation between the compound score of app usage frequency and baseline and follow-up information

Variables*	Spearman coefficient (95%CI)	*P* Value
Age, year	-0.05 (-0.18, 0.08)	0.469
Age at onset, year	-0.05 (-0.19, 0.08)	0.458
Disease duration, year	-0.05 (-0.18, 0.09)	0.473
BMI, kg/m^2	0.00 (-0.17, 0.17)	0.959
Education, year	0.04 (-0.17, 0.24)	0.718
LED, mg/day	0.14 (0.00, 0.27)	0.047
Baseline self-evaluation		
PDQ-8 score	-0.05 (-0.18, 0.08)	0.471
GDS-15 score	-0.13 (-0.32, 0.07)	0.210
MDS-UPDRS IB score	-0.08 (-0.25, 0.09)	0.339
MDS-UPDRS II score	-0.03 (-0.20, 0.14)	0.729
1-year follow-up self-evaluation		
PDQ-8 score	-0.15 (-0.27, -0.02)	0.026
GDS-15 score	-0.27 (-0.48, -0.03)	0.028
MDS-UPDRS IB score	-0.26 (-0.49, 0.01)	0.062
MDS-UPDRS II score	-0.36 (-0.58, -0.10)	0.008

*

Sample sizes (N=226)

Spearman rank correlation was used to assess the correlation. Spearman coefficient is used to calculate the effect size.

Abbreviations: 95% CI = 95% Confidence Interval; BMI = body mass index; LED = levodopa equivalent dose; PDQ-8 = Parkinson Disease Questionnaire 8; GDS-15 = Geriatric Depression Scale 15; MDS-UPDRS = Movement Disorder Society-Sponsored Revision of the Unified Parkinson's Disease Rating Scale.
