# Supplementary material for: Heath management app use in Parkinson’s disease and quality of life during the COVID-19 pandemic
Source: Commun Med (Lond). 2023 Feb 11;3:23. doi: 10.1038/s43856-023-00246-4 (PMC9919748; doi:10.1038/s43856-023-00246-4)
Supplement: Supplementary file 2 — Reporting Summary [file 43856_2023_246_MOESM2_ESM.pdf]

nature portfolio

Corresponding author(s): Jianjun Wu, Jian Wang

Last updated by author(s): Jan 16, 2023

Reporting Summary

Nature Portfolio wishes to improve the reproducibility of the work that we publish. This form provides structure for consistency and transparency in reporting. For further information on Nature Portfolio policies, see our Editorial Policies and the Editorial Policy Checklist.

Statistics

For all statistical analyses, confirm that the following items are present in the figure legend, table legend, main text, or Methods section.

n/a	Confirmed	
☐	☒	The exact sample size (*n*) for each experimental group/condition, given as a discrete number and unit of measurement
☐	☒	A statement on whether measurements were taken from distinct samples or whether the same sample was measured repeatedly
☐	☒	The statistical test(s) used AND whether they are one- or two-sided<br>*Only common tests should be described solely by name; describe more complex techniques in the Methods section.*
☐	☒	A description of all covariates tested
☐	☒	A description of any assumptions or corrections, such as tests of normality and adjustment for multiple comparisons
☐	☒	A full description of the statistical parameters including central tendency (e.g. means) or other basic estimates (e.g. regression coefficient) AND variation (e.g. standard deviation) or associated estimates of uncertainty (e.g. confidence intervals)
☐	☒	For null hypothesis testing, the test statistic (e.g. *F*, *t*, *r*) with confidence intervals, effect sizes, degrees of freedom and *P* value noted<br>*Give P values as exact values whenever suitable.*
☒	☐	For Bayesian analysis, information on the choice of priors and Markov chain Monte Carlo settings
☒	☐	For hierarchical and complex designs, identification of the appropriate level for tests and full reporting of outcomes
☐	☒	Estimates of effect sizes (e.g. Cohen's *d*, Pearson's *r*), indicating how they were calculated

Our web collection on statistics for biologists contains articles on many of the points above.

Software and code

Policy information about availability of computer code

Data collection	Pawei APP
Data analysis	The data analysis was conducted by SAS 9.4 (SAS Institute Inc., Cary, NC, USA).

For manuscripts utilizing custom algorithms or software that are central to the research but not yet described in published literature, software must be made available to editors and reviewers. We strongly encourage code deposition in a community repository (e.g. GitHub). See the Nature Portfolio guidelines for submitting code & software for further information.

Data

Policy information about availability of data

All manuscripts must include a data availability statement. This statement should provide the following information, where applicable:

- Accession codes, unique identifiers, or web links for publicly available datasets
- A description of any restrictions on data availability
- For clinical datasets or third party data, please ensure that the statement adheres to our policy

We can share our relevant raw data supporting our findings. If any scientist wish to use them for non-commercial purposes, without breaching participant confidentiality, he/her can contact us directly, and we will share our raw data freely with he/her.

Human research participants

Policy information about studies involving human research participants and Sex and Gender in Research.

Reporting on sex and gender	In Chinese culture, sex (biological attribute) and gender (shaped by social and cultural circumstances) were consistent, so the disaggregated sex and gender data wasn't collected. Sex- and gender-based analyses weren't performed. Gender was determined based on self-reporting, and our findings applied to gender.
Population characteristics	See below.
Recruitment	PD patients who owned IOS or Android smartphones were invited to download an Pawei APP. After completing the registration, the app directs the participants to an informed consent page, where the participants can either "agree" or "decline" their participation. First, participants were people who owned IOS or Android smartphones and thus were not representative of the broader PD population. Second, the present observation lasted for one year; although this is reasonable for a first orienting studies such as ours, more work remains needed to monitor the effect of a smartphone-based remote management app in the long term.
Ethics oversight	This study approved by the Human Studies Institutional Review Board, Huashan Hospital, Fudan University.

Note that full information on the approval of the study protocol must also be provided in the manuscript.

Field-specific reporting

Please select the one below that is the best fit for your research. If you are not sure, read the appropriate sections before making your selection.

☐ Life sciences ☒ Behavioural & social sciences ☐ Ecological, evolutionary & environmental sciences

For a reference copy of the document with all sections, see nature.com/documents/nr-reporting-summary-flat.pdf

Behavioural & social sciences study design

All studies must disclose on these points even when the disclosure is negative.

Study description	qualitative cohort study
Research sample	Participants were Chinese patients with Parkinson's disease who registered in Pawei APP. Demographic information included age, age at onset, disease duration, BMI, education, gender, custom hand, PDQ-8, GDS-15, MDS-UPDRS IB, MDS-UPDRS II, and levodopa equivalent dose. The sample is not representative.
Sampling strategy	The sampling procedure was convenient. No sample-size calculation was performed. All participants conformed to the criteria were included in this study.
Data collection	PD patients who owned IOS or Android smartphones were invited to download an Pawei APP. After completing the registration, the app directs the participants to an informed consent page, where the participants can either "agree" or "decline" their participation. Following consent, participants are asked to complete self-evaluation questionnaires, at baseline and at least once each season (at 90-day intervals) for regular evaluation. The researcher was blind during data collection.
Timing	The start and stop dates of data collection was Jan 20, 2019 and Oct 6, 2020, separately. There is no gap for collection periods.
Data exclusions	4037 PD patients were excluded, and the exclusion reasons were shown in eFigure 1.
Non-participation	706 PD patients dropped out, because they didn't complete 1-year follow-up self-evaluation.
Randomization	Allocation was not random. Age, gender, education, disease duration, and levodopa equivalent dose were controlled.

Reporting for specific materials, systems and methods

We require information from authors about some types of materials, experimental systems and methods used in many studies. Here, indicate whether each material, system or method listed is relevant to your study. If you are not sure if a list item applies to your research, read the appropriate section before selecting a response.

Materials & experimental systems

n/a	Involved in the study	
☒	☐	Antibodies
☒	☐	Eukaryotic cell lines
☒	☐	Palaeontology and archaeology
☒	☐	Animals and other organisms
☐	☒	Clinical data
☒	☐	Dual use research of concern

Methods

n/a	Involved in the study	
☒	☐	ChIP-seq
☒	☐	Flow cytometry
☒	☐	MRI-based neuroimaging

Clinical data

Policy information about clinical studies

All manuscripts should comply with the ICMJE guidelines for publication of clinical research and a completed CONSORT checklist must be included with all submissions.

Clinical trial registration	The study is registered at the ClinicalTrials.gov under registration number NCT03649503.
Study protocol	The full trial protocol can be accessed through this website: https://clinicaltrials.gov/ct2/show/NCT03649503?cond=NCT03649503&draw=2&rank=1.
Data collection	Our data was collected by Pawei APP. The time periods of recruitment and data collection was Jan 20, 2019 and Oct 6, 2020, separately. There is no gap for collection periods.
Outcomes	The primary outcome was change from baseline in the Short-Form 8-Item Parkinson's Disease Questionnaire (PDQ-8) Score assessed by patients themselves through Pawei APP. The secondary outcome was GDS-15 change score, MDS-UPDRS IB change score, and MDS-UPDRS II change score assessed by patients themselves through Pawei APP.
